# Disrupting Sleep: The Effects of Sleep Loss on Psychotic Experiences Tested in an Experimental Study With Mediation Analysis

**DOI:** 10.1093/schbul/sbx103

**Published:** 2017-08-04

**Authors:** Sarah Reeve, Richard Emsley, Bryony Sheaves, Daniel Freeman

**Affiliations:** 1 Department of Psychiatry, University of Oxford, Oxford, UK; 2 Centre for Biostatistics, University of Manchester, Manchester Academic Health Science Centre, Manchester, UK

**Keywords:** insomnia, manipulation, psychosis, paranoia, hallucinations, grandiosity, cognitive disorganization, schizophrenia, mechanisms

## Abstract

Our view is that insomnia may be a causal factor in the occurrence of psychotic experiences such as paranoia and hallucinations. However, the causal relationship is not established. The aim of the study was to investigate the causal role of insomnia in psychotic experiences via a sleep restriction manipulation. The study was a within-subjects crossover design that included a planned mediation analysis. Sixty-eight nonclinical volunteers underwent a sleep loss condition (restricted to 4 h sleep for 3 nights) and a control condition (standard sleep) in randomized order in 2 consecutive weeks, with a weekend washout period. Psychotic experiences (paranoia, hallucinations, grandiosity, and cognitive disorganization) and candidate mediating variables (negative affect and related processes, working memory, decision making, and perceptual processing) were assessed before and after each condition. Actigraphy verified an average sleep duration of 5 h 15 min in the sleep loss condition, vs 6 h 58 min in the control condition. After the sleep loss condition, relative to the control condition, participants reported significant increases in paranoia, hallucinations, and cognitive disorganization, with no significant changes in grandiosity. The sleep loss condition was also associated with significant increases in negative affect, negative self and other cognitions, worry, and working memory impairment. Mediation analyses indicated that changes in psychotic experiences were mediated by changes in negative affect and related processes, but not memory impairment. The overall conclusion is that insomnia has a causal role in the occurrence of certain psychotic experiences, and that a key route is via negative affect.

## Background

Insomnia co-occurs with and predicts psychotic experiences, especially paranoia and hallucinations. In a recent survey of 267692 individuals from 56 countries, sleep problems were associated with a 2-fold increased likelihood of endorsing that there was a plot to harm them (paranoia) or an experience of seeing visions or hearing voices (hallucinations).^1^ Insomnia also predicts an increased likelihood of reporting paranoia or hallucinations at a later time.^2,3^ These associations remained significant even after controlling for depression and anxiety. In clinical settings sleep difficulties co-occur with more severe psychotic symptoms in patients with schizophrenia spectrum disorders,^4–7^ are predictive of relapse of psychotic symptoms,^8,9^ and for transition from at risk mental state to first-episode psychosis.^10^

Clearly, there is an association between insomnia and psychotic experiences. Yet, as identified by a recent systematic review of 66 studies, there is a need to directly test the causal role of insomnia, and to understand the mechanisms linking it with psychotic experiences.^11^ Such findings would inform the understanding of the etiology of psychotic experiences, and would also contribute to developing sleep interventions for patients with non-affective psychosis.^12^ To establish causation, studies are needed which manipulate the proposed causal variable (insomnia) to test the effect (on psychotic experiences).^13^ One approach is to improve insomnia while measuring psychotic experiences among individuals with existing sleep problems (with or without a psychotic disorder diagnosis).^12,14^ An improvement in sleep should lead to a lessening in psychotic experiences. A second approach is to disrupt sleep, with the prediction that psychotic experiences should worsen. In effect, with nonclinical individuals, this would temporarily simulate the development of psychotic experiences.

Table 1 lists the 3 sleep disruption studies measuring psychotic experiences reported to date.^15–17^ These studies are consistent with the idea that sleep loss results in an increase in psychotic experiences. However, the findings in relation to individual psychotic experiences are inconsistent. For example, paranoia is increased following sleep deprivation in Kahn-Green et al^15^ but not Petrovsky et al.^17^ Furthermore, these studies manipulate sleep via total sleep deprivation, whereas insomnia is characterized by insufficient sleep over a period of time. Lastly, none of these studies included measurement or analysis of potential mediating factors.

**Table 1.** Existing Sleep Disruption Studies Addressing Psychotic Experiences

Citation	*N*	Sample Characteristics	Psychotic Experience Measure	Sleep Manipulation	Findings
Hurdiel et al^16^	17	Volunteers completing ultramarathon event	Hallucinations—open ended question on completion	*Sleep deprivation*	4 out of 17 participants reported experiencing hallucinations during the exercise event
				Average 46 h 38 min (measured by actigraphy)	
Kahn-Green et al^15 ^	25	Nonclinical volunteers (recruited from military)	PAI (pre- and postsleep deprivation)	*Sleep deprivation*	Sleep deprivation resulted in an increase in anxiety, depression, and paranoia, but not manic-related symptoms or schizophrenia symptom factors
				56 h (in lab)	
Petrovsky et al^17 ^	24	Nonclinical volunteers (Students)	PSI (pre- and postsleep deprivation)	*Sleep deprivation*	Sleep deprivation induced perceptual distortions, cognitive disorganization, and anhedonia, but not mania, paranoia, or delusional thinking
				Overnight sleep deprivation (in lab)	

*Note*: PAI, Personality Assessment Inventory^18 ^; PSI, Psychotomimetic States Inventory.^19^

Cognitive models of psychotic experiences suggest potential means by which insomnia leads to psychotic experiences. Negative affect has been found to explain a proportion of the relationship between sleep dysfunction and psychotic experiences.^11^ This would be predicted by models of paranoia and hallucinations which place anxiety and depression in the center of development and maintenance of positive psychotic symptoms.^20–22^ There are other untested factors which could mediate the relationship between sleep dysfunction and psychosis. Working memory deficits are a commonly noted consequence of sleep loss and insomnia^23,24^ and have been linked to the jumping to conclusions reasoning bias,^25^ which is itself a key component of cognitive models for delusions.^20^ Changes in top–down perceptual processing (ie, influence of beliefs on sensory processing) have also been reported following sleep loss, with sleep deprivation causing individuals to be less susceptible to the effects of visual or auditory illusions.^26^ A similar effect of reduced susceptibility to illusions has been found in individuals with schizophrenia.^27^ Furthermore, changes in top-down perceptual processing are a common feature in cognitive models of hallucinations.^22^ Therefore, there is a strong theoretical basis for testing the contribution of mediating factors such as affect-related processing, working memory, perceptual processing, and decision making in the relationship between insomnia and psychotic experiences.

### The Current Study

The current study aims to investigate the causal role of insomnia in psychosis by assessing the effect of sleep loss on psychotic experiences and mediating variables in a nonclinical sample.

In our sleep loss condition, sleep was restricted to 4 h sleep each night for 3 consecutive nights. A review of the literature indicated that this would be appropriate for causing noticeable changes, while still being reversible in a weekend washout period.^28–32^ This sleep restriction was self-enforced by participants at home, and they continued their daily activities (study, work, etc.) during the study. Adherence was monitored by actigraphic recording, responses to text messages (SMS) prompts, and sleep diaries. Sleep was restricted by participants delaying their sleep onset; in other studies modeling insomnia (eg, by Finan et al^33,34^) sleep has been restricted by waking participants during the night. However, this is challenging to administer outside of a sleep laboratory, which in turn limits ecological validity.

We tested the following hypotheses to establish a causal relationship between the sleep loss condition and psychotic experiences and to test the role of mediating factors in this relationship.

1. Sleep loss will cause an increase in (subclinical) psychotic experiences.
2. Sleep loss will cause changes in mood and cognition that may underlie the development of psychotic experiences.
3. Changes in mood and cognition will mediate the changes in psychotic experiences.

## Recruitment

Eighty-five participants consented to take part, of which 75 completed the study. Seven participants were excluded for noncompletion or late completion of an assessment, resulting in a final sample of 68 on which all analyses were carried out. Participants were recruited via advertisements from the student and wider community in Oxford. Participants were aged 18–30 to reduce confounding by changes in sleep with age.^35^ This is also the age at which psychotic symptoms generally emerge for those who go on to receive a diagnosis of the clinical disorder.

Other inclusion criteria were good quality sleep (scoring ≤5 on the Pittsburgh Sleep Quality Index^36^), no history of psychiatric disorder, and no current mood disorder (indicated by scoring below clinical cutoff on the Beck Depression Inventory^37^ and the Mood Disorder Questionnaire^38^). The exclusion criteria were travel across time zones in the preceding 2 weeks, employment requiring shift work, consumption of drugs or medication (besides contraceptives or vitamins), requirement to drive, cycle, or operate heavy machinery during the study, and nonfluency in English. The study received approval from the local University Research Ethics Committee, and written informed consent was received from all participants.

## Study Design

A within subjects cross-over design was chosen. Following consent participants were randomized to the sleep loss condition (4 h sleep a night for 3 nights) or the control condition (standard sleep) for their first week in the study, with the alternate condition carried out in the second week. Both conditions were carried out Monday–Thursday or Tuesday–Friday, with a minimum 3 night washout period over Friday, Saturday, and Sunday nights. Assessments were carried out at the start and end of both conditions. A diagram of the study design is included in the supplementary material.

### 

#### Sleep Loss Condition (Restricted Sleep)

Participants set their own 4 h sleep period based on delaying sleep time while keeping wake time constant. For example, a participant who normally slept from 11:00 pm until 07.00 am would sleep from 03:00 am to 07.00 am each night in the restricted condition. Participants responded to automated hourly text (SMS) messages to demonstrate they were awake. The messages were sent from 10.00 pm until the hour before the participant’s chosen sleep time.

#### Control Condition (Standard Sleep).

In this condition, participants could sleep however they wished for the 3 nights and were not required to respond to any text messages.

A self-report sleep diary based on the Consensus Sleep Diary^39^ was completed in both study conditions. Objective recording of sleep-wake patterns was conducted in both sleep conditions using wrist actigraphy (CamNTech MotionWatch 8). MotionWare software was used to provide an estimate of total sleep time in each 24 h period. Alcohol, caffeine, and nicotine consumption was recorded in the diary.

### Assessments

All assessments were administered online via the Inquisit platform.^40^ Assessments were completed between 1800 h and 0000 h. The timescale of all questionnaires was adjusted to the previous 3 days to allow for repeated administration. Higher scores in all measures indicate greater severity. The assessment battery comprised:

#### Psychotic Experiences.

Specific Psychotic Experiences Questionnaire^41^: The SPEQ is a self-report questionnaire with 5 dimensions (paranoia, hallucinations, cognitive disorganization, grandiosity, and anhedonia). The outcome measure is the total score in each dimension. Distress is rated for each dimension, the average of these ratings is an additional outcome measure. The anhedonia subscale was removed for this study as the items were unsuitable for repeated administration.

#### Negative Affect.

Depression Anxiety Stress Scale (short form)^42^: The DASS is a 21-item questionnaire with 3 dimensions (depression, anxiety, and stress). Each item is marked on a 0–3 agreement scale. The outcome variables are the total score for each dimension.

#### Affective Processes.

Worry questionnaire (Freeman et al, in preparation): 10 statements ranked on 5-point scale for agreement (0–4). Items include “There was little I could do to stop worrying” and “Worry has caused me to feel upset.” The outcome variable is the total sum of responses.

Brief core schema scale^43^: The BCSS is a 24 item scale assessing beliefs about self and others. The participant rates each belief on a 0–4 agreement scale. Outcome variables are the total score within each subscale (negative self, positive self, negative other, positive other).

#### Perception.

Ebbinghaus illusion task: participants decide which of 2 orange circles are larger. Grey surrounding circles present a misleading illusory effect on a third of trials, a helpful illusory effect on another third, and there are no surrounding circles in the remainder of trials (control trials). The outcome variable is the average of the absolute difference between each illusory condition and the control condition. The current task was modeled on that used by Doherty et al.^44^

Perceptual vigilance task^45^: Participants click as soon as possible when a red dot appears in 1 of 9 locations on screen (at intervals randomly chosen from 1 to 10 s) over 5 min. Outcome variables were number of lapses (≥500 ms response time) and average reaction time.

#### Decision Making.

Jumping to conclusions^46^ task: The 85:15 version of the task was used. Participants requested “randomly selected” beads one at a time with replacement until they were certain which jar the beads were from (the beads are actually in a predetermined sequence). The outcome variable is the number of beads taken to make a decision.

#### Working Memory.

N-Back task^47^: Participants are presented with a sequence of letters, and click when the current letter matches the letter 1 position, 2 position, or 3 positions back in the sequence. Participants completed 2 blocks of each of the 1, 2, and 3 back tasks in a random order. Each block contained 20 letters, with a 1/3 rate of matching letters. The outcome variable is the decision value, calculated by the below formula:

$$\text{Decision value}=\frac{\text{Correct hits}-\text{False alarms}}{\text{Total number of trials}}$$

The minimum value is 0, maximum score is 0.3.

### Statistical Analysis

Data for this 2-condition 2-period crossover trial were analyzed using Stata version 14.^48^ Demographic and adherence data were analyzed using SPSS 22.0.^49^ There were no missing data. Statistical significance was set at *P* <.05.

Actigraphic and sleep diary estimates of sleep, and nicotine, alcohol, and caffeine consumption were checked for adherence using paired *t*-tests.

The primary data analysis was performed using multilevel modeling, to account for the repeated measures within an individual and the 2 measures of “baseline” before each of the sleep conditions. For each outcome, we fitted a linear mixed model with a fixed effect for order, the sleep condition and the “baseline” measure of the outcome, with a random intercept for each participant. We repeated this modeling for each putative mediator as an outcome. Effect sizes were calculated using the first baseline standard deviation (pooled across conditions and order groups) as the denominator,^50^ which can be interpreted as equivalent to Cohen’s *d*.

Although there has been considerable discussion of the underlying assumptions in statistical mediation analysis,^51^ a crossover experimental design is a scenario in which these assumptions will plausibly hold. If an effect of the sleep condition on both mood or cognition (the putative mediators) and subclinical psychotic symptomatology (the final outcome) is demonstrated, then we evaluated mediation using the strategy suggested by Baron and Kenny.^52^ In the final mediation analysis, we only examine mediators and outcomes with a significant condition effect (*P* < .05) from the previous analysis. For each significant outcome, this involved fitting the additional linear mixed model as above with the addition of the putative mediators at “baseline” and end of intervention as fixed effects. The total effect is the effect of sleep condition on outcome in the first model. The direct effect is the effect of sleep condition on outcome in the model including the mediators. The indirect effect is the product of the coefficient of sleep condition on the mediator, and the coefficient of the mediator on outcome. All three models were bootstrapped with 1000 replications to obtain valid standard errors and significance tests. If the direct effect is smaller than the total effect, it becomes nonsignificant in the presence of the mediator, and the indirect effect itself is statistically significant, then we would conclude mediation. As each mediator is considered individually, we believe our sample size provides sufficient power to detect indirect effects. The proportion mediated was calculated as the ratio of the indirect effect to the total effect.

## Results

### Demographics

The demographic data for the participants are presented in table 2. The 68 study participants were balanced with respect to gender (54% female), were predominantly white British (69%), and the majority (76%) were undergraduate or postgraduate students.

**Table 2.** Demographics of Study Sample

	Total Sample	First Week Restricted	Second Week Restricted
	*N* = 68	(*n* = 33)	(*n* = 35)
Mean age in years (SD)	22.5 (3.4)	21.9 (3.1)	23.0 (3.7)
Gender			
Male	31 (45.6%)	14 (42.4%)	17 (48.6%)
Female	37 (54.4%)	19 (57.6%)	18 (51.4%)
Ethnicity			
White/White British	47 (69.1%)	22 (66.7%)	25 (71.4%)
Asian/Asian British	12 (17.6%)	6 (18.1%)	6 (17.1%)
Mixed/multiple ethnic groups	5 (7.4%)	1 (3.0%)	4 (11.4%)
Black/African/Caribbean/Black British	2 (2.9%)	2 (6.1%)	0 (0.0%)
Other	2 (2.9%)	2 (6.1%)	0 (0.0%)
Occupation			
Undergraduate student	33 (48.5%)	17 (51.5%)	16 (45.7%)
Postgraduate student	19 (27.9%)	9 (27.3%)	10 (28.6%)
Part time employed	5 (7.4%)	2 (6.1%)	3 (8.6%)
Full time employed	10 (14.7%)	5 (15.2%)	5 (14.3%)
Unemployed	1 (1.5%)	0 (0.0%)	1 (2.9%)

### Adherence to Sleep Restriction

Nine hundred seventy-seven of the 993 SMS messages (98.4%) sent to the participants were responded to within the 15-min window. The total sleep time recorded in the diaries (3 h 54 min sleep loss condition vs 7 h 21 min control condition) and by actigraphy (5 h 15 min vs 6 h 58 min) show a significant reduction in sleep time (both *P* < .001). No significant difference was found in alcohol (0.81 units vs 1.02 units, *P* = .223) or nicotine (1.47 mg vs 1.26 mg, *P* = .211) intake across the 2 weeks. A slight but significant increase in caffeine intake was recorded in the sleep loss condition (104.32 mg vs 87.23 mg, *P* = .006).

### Changes in Outcomes by Condition

#### Psychotic Experiences.

In table 3, it can be seen that, in comparison to the control condition, the sleep loss condition was associated with significant increases in hallucinations, paranoia, and cognitive disorganization. Distress from psychotic experiences also significantly increased relative to the control condition. All these changes had moderate to large effect sizes (ranging from *d* = 0.38 to *d* = 0.87). However, no significant changes due to sleep condition were found on grandiosity. There was a significant interaction between baseline scores and the sleep condition effect in paranoia (effect = 0.535 [SE = 0.156], *P* = .001), hallucinations (1.228 (0.280), *P* < .001), and psychotic experience distress (0.388 (0.154), *P* = .012), indicating that a higher baseline score resulted in a proportionally higher sleep condition effect. Further details can be found in our supplementary material.

**Table 3.** Changes in Psychotic Experiences and Candidate Mediators Following Sleep Restriction Vs Control (Standard Sleep) Condition

	Sleep	Baseline Mean (SD)	Endpoint Mean (SD)	Effect (SE), *P*-Value	95% CI	Effect Size
Psychotic experiences						
Paranoia (SPEQ)	Restricted	2.3 (3.4)	3.8 (5.4)	1.57 (0.54), .003	2.63, −0.52	0.383
	Standard	2.8 (4.7)	2.5 (5.4)			
Hallucinations (SPEQ)	Restricted	0.4 (0.8)	1.2 (2.4)	1.06 (0.28). .000	1.62, −0.51	0.869
	Standard	0.3 (1.5)	0.1 (0.7)			
Cognitive Disorganization (SPEQ)	Restricted	2.6 (3.9)	4.8 (4.2)	2.44 (0.36), .000	3.14, −1.73	0.643
	Standard	2.7 (3.8)	2.5 (3.5)			
Grandiosity (SPEQ)	Restricted	6.6 (6.7)	6.7 (6.6)	0.14 (0.52), .780	1.16, −0.87	0.002
	Standard	6.4 (6.1)	6.4 (6.5)			
Psychotic experience distress (SPEQ)	Restricted	0.3 (0.8)	0.7 (1.1)	0.42 (0.12), .001	66, −0.18	0.521
	Standard	0.4 (0.8)	0.3 (0.7)			
Mediating variables						
Depression (DASS)	Restricted	2.0 (3.9)	4.6 (6.1)	2.80 (0.61), .000	3.99, −1.61	0.776
	Standard	1.8 (3.2)	1.6 (3.9)			
Anxiety (DASS)	Restricted	2.2 (3.2)	5.2 (5.2)	3.53 (0.67), .000	4.84, −2.22	1.002
	Standard	3.0 (3.8)	2.1 (3.9)			
Stress (DASS)	Restricted	3.8 (6.2)	7.8 (7.8)	4.61 (0.85), .000	6.27, −2.95	0.806
	Standard	4.0 (5.3)	3.3 (4.6)			
Negative beliefs about self (BCSS)	Restricted	1.8 (2.2)	2.6 (3.2)	1.05 (0.28), .000	1.60, −0.50	0.463
	Standard	1.9 (2.3)	1.6 (2.1)			
Positive beliefs about self (BCSS)	Restricted	13.1 (5.3)	12.0 (5.1)	−0.55 (0.53), .305	0.50, −1.59	0.113
	Standard	13.7 (4.4)	130 (5.3)			
Negative beliefs about others (BCSS)	Restricted	1.9 (2.7)	2.6 (3.7)	0.99 (0.36), .006	1.68, −0.29	0.363
	Standard	2.0 (2.8)	1.7 (2.4)			
Positive beliefs about others (BCSS)	Restricted	12.0 (5.0)	11.8 (4.9)	0.19 (0.56), .737	1.28, −0.91	0.044
	Standard	12.9 (3.7)	12.1 (5.0)			
Worry (Worry scale)	Restricted	5.9 (6.4)	7.6 (7.3)	2.59 (0.92), .005	4.39, −0.80	0.402
	Standard	6.0 (6.6)	5.0 (6.0)			
Ebbinghaus—difference in scores	Restricted	40.3 (9.3)	39.7 (11.0)	0.74 (1.19), .538	3.06, −1.60	0.079
	Standard	40.8 (9.2)	39.4 (10.2)			
Perceptual vigilance—average RT (ms)	Restricted	603.5 (975.3)	613.2 (409.9)	63.13 (56.11), .261	173.10, −46.84	0.233
	Standard	510.7 (300.8)	539.0 (283.7)			
Perceptual vigilance—no. of lapses (RT ≤ 500 ms)	Restricted	12.0 (12.9)	18.1 (15.5)	2.65 (1.48), .072	5.56, −0.24	0.214
	Standard	11.1 (11.8)	15.4 (12.4)			
Working memory (*N* back decision value)	Restricted	0.2 (0.1)	0.2 (0.1)	−0.03 (.01), .005	−0.01, −0.04	0.545
	Standard	0.2 (0.0)	0.2 (0.1)			
Jumping to conclusions (no. of. beads)	Restricted	3.5 (4.1)	4.6 (4.5)	0.02 (0.27), .925	0.55, −0.50	0.005
	Standard	4.2 (3.6)	4.3 (3.5)			

#### Mediators.

Highly significant and large effect size increases were found for depression, anxiety, and stress following the sleep loss condition, as compared with the control condition. We also found significant and moderate effect size increases in worry and in the endorsement of negative beliefs about self and others following sleep loss. There were no significant changes in endorsements of positive beliefs about self or others.

Working memory task performance significantly worsened following the sleep loss condition as compared with the control condition (effect = 0.55). No significant changes were seen in the number of beads requested before decision in the jumping to conclusions task. There were no significant changes in the illusion effect in the Ebbinghaus task. There was no significant effect of sleep condition on vigilance (reaction time or lapses).

### Mediation

The outcome of mediation analyses can be seen in table 4. The effect of sleep loss on paranoia is almost completely mediated by negative affect (all >90%). Affective processes, in particular, negative beliefs about others, also mediated a proportion of the effect. Working memory change was not a significant mediator for paranoia or for any other psychotic experience (ie, the indirect effect via working memory did not reach significance for any outcome). The results for cognitive disorganization are similar to those for paranoia, with significant mediation by negative affect, negative beliefs about self, and worry. However, the proportion mediated by these factors (26.2–39.2%) was smaller than for paranoia. The only significant mediator found for hallucinations was stress (43.4%). Distress from psychotic experiences was again predominantly mediated by negative affect (51.4%–57% across dimensions), with no other mediators reaching significance.

**Table 4.** Mediation Analysis for Increases in Psychotic Experiences

Outcome	Total Effect, Estimate (SE), *P* Value	Putative Mediator	Direct Effect, Estimate (SE), *P* Value	Indirect Effect, Estimate (SE), *P* Value	Proportion Mediated %
SPEQ paranoia	−1.57 (0.59), .008	Anxiety	−0.26 (0.79), .747	−1.44 (0.59), .014	91.7
		Depression	0.12 (0.54), .816	−1.45 (0.42), .001	92.1
		Stress	−0.02 (0.63), .969	−1.55 (0.51), .002	98.7
		Neg self	−1.13 (0.50), .023	−0.43 (0.33), .195	27.2
		Neg other	−0.92 (0.54), .084	−0.64 (0.24), .009	40.8
		Worry	−0.86 (0.73), .238	−0.67 (0.39), .082	42.7
		Working memory	−1.51 (0.66), .022	−0.07 (0.18), .687	4.7
SPEQ hallucinations	−1.06 (0.29), <.001	Anxiety	−0.88 (0.28), .002	−0.23 (0.18), .193	22.0
		Depression	−0.89 (0.32), .005	−0.13 (0.12), .276	12.9
		Stress	−0.61 (0.22), .006	−0.46 (0.23), .045	43.4
		Neg self	−0.81 (0.27), .002	−0.27 (0.16), .094	25.4
		Neg other	−0.93 (0.28), .001	−0.14 (0.08), .071	12.7
		Worry	−0.93 (0.30), .002	−0.14 (0.12), .200	13.4
		Working memory	−1.06 (0.30), <.001	−0.01 (0.07), .912	0.7
SPEQ cognitive disorganization	−2.44 (0.40), <.001	Anxiety	−1.52 (0.41), <.001	−0.96 (0.31), .002	39.2
		Depression	−1.78 (0.40), <.001	−0.64 (0.25), .011	26.2
		Stress	−1.55 (0.40), <.001	−0.90 (0.28), .001	36.8
		Neg self	−2.05 (.38), <.001	−0.41 (.18), .026	16.8
		Neg other	−2.26 (0.37), <.001	−0.17 (0.15), .242	7.0
		Worry	−1.90 (0.36), <.001	−0.53 (0.26), .042	21.7
		Working memory	−2.40 (0.43), <.001	−0.03 (0.09), .706	1.4
SPEQ distress	−0.42 (0.14), .002	Anxiety	−0.18 (0.13), .174	−0.24 (0.12), .048	56.9
		Depression	−0.17 (0.12), .145	−0.24 (0.09), .009	57.0
		Stress	−0.20 (0.12), .080	−0.22 (0.09), .022	51.4
		Neg self	−0.27 (0.12), .020	−0.15 (0.08), .056	34.8
		Neg other	−0.37 (0.13), .005	−0.05 (0.03), .111	12.3
		Worry	−0.28 (0.12), .022	−0.14 (0.08), .082	33.3
		Working memory	−0.34 (0.14), .012	−0.08 (0.06), .162	18.4

## Discussion

This is the first study to test the etiology of psychotic experiences by simulating insomnia-like sleep loss in nonclinical volunteers. Participants adhered to the self-enforced sleep restriction, as demonstrated by the SMS responses and actigraphy. Sleep loss resulted in moderate to large effect size increases in paranoia, hallucinations, and cognitive disorganization as compared to standard sleep. There was no change in grandiosity. The significant interaction between baseline psychotic experience scores and sleep condition indicates a greater effect of sleep restriction on those endorsing more psychotic experiences at baseline. Large increases in depression and anxiety were found following the sleep loss condition (compared with the control condition), along with moderate increases in the endorsement of negative beliefs about self and others, and in worry. There were no significant changes in positive beliefs about self or others. Significant impairment in working memory was found following the sleep loss condition. However, no changes were found for perceptual processing (illusory effects or vigilance), or decision making. Overall the results are consistent with a causal role for insomnia in certain psychotic experiences.

The lack of change in grandiosity could be understood within the context of the large increase in negative affect. Emotion congruent models of grandiosity would predict that negative affective states are not conducive to the development of these beliefs.^53^ The absence of an effect of restricted sleep on perceptual vigilance is notable, given this is a common consequence of sleep restriction in previous laboratory-based studies.^30–32^ In the current study, vigilance decreased across the week in both sleep conditions. While there was a greater decrease in vigilance in the sleep loss condition, a social jet lag effect in the control condition may have reduced the contrast with the sleep loss condition.^54^ The lack of change in jumping to conclusions bias suggests that this is not a route by which reduced sleep leads to increased psychotic experiences.

The mediation analysis sheds light on the link between disrupted sleep and psychotic experiences. This shows that negative affect is by far the most relevant factor in explaining the effect of sleep disruption on psychotic experiences. These findings join others in demonstrating the importance of negative affect in this relationship (in both clinical and nonclinical populations).^55,56^ Conversely, despite there being significant deficits in working memory following sleep loss, these changes did not mediate any significant proportion of the effect on psychotic experiences.

This is especially the case for paranoia, where 90% or more of the relationship was mediated by depression, anxiety, or stress. This pattern is identical to that found in a previous study by our group,^57^ which tested the mechanisms underlying paranoia by the administration of ∆9-tetrahydrocannabinol (the principal psychoactive ingredient of cannabis). As in the current study this induced increases in negative affect, working memory impairment and paranoia. The increased negative affect mediated the relationship between the experimental manipulation and increased paranoia, with no significant role for the working memory impairment. The significant mediation by both worry and negative self-belief in our study is also interesting in the context of clinical trials demonstrating that addressing these affective processes can improve symptoms of paranoia in individuals with persecutory delusions.^58,59^ These results emphasize the key role of negative affect and affective cognitions in paranoia,^60^ above neuropsychological factors.

The increase in hallucinations was less well accounted for by the mediators tested. Only one aspect of negative affect (stress) was identified as a possible mediator. This implies that different mechanistic factors may link sleep dysfunction to hallucinations, supporting the need to research and measure psychotic experiences individually rather than grouping together as a unitary “psychosis.”^18,19,61^

### Limitations and Future Directions

Our study models insomnia by delaying sleep onset to decrease total sleep time. However, insomnia is also characterized by decreases in sleep efficiency (more time spent in bed awake) and decreased sleep continuity (waking during the night), which are not simulated in our manipulation. Nevertheless, we believe that the ecological validity of our design, wherein participants were required to go about their daily lives while suffering from the consequences of reduced sleep, offers a significant advantage in allowing us to replicate some aspects of insomnia as experienced by the clinical population. It may also be worth considering that a study comparing delayed onset vs disrupting continuity found that continuity disruptions resulted in even greater increases in negative affect than delayed sleep onset.^33^ Therefore, the current study may underestimate the impact of insomnia on mood and psychotic experiences.

It is worth mentioning that the actigraphic recording indicated incomplete adherence to the sleep restriction (5 h 15 of sleep, rather than 4 h). The analysis algorithm assumes periods of inactivity amount to sleep, therefore sleep can be overestimated when individuals are tired and less active. Regardless, as the study aimed to investigate the effects of reduced sleep (rather than precisely the effect of 4 h of sleep), the conclusions of the study remain.

Psychotic experiences were generally endorsed at a low level in our sample, and all changes remained in the nonclinical range. However, it should be noted that this was screened to be a “psychologically healthy” sample and that this was a mild sleep restriction manipulation. Furthermore, our interaction analysis shows that those endorsing more psychotic experiences at baseline had a greater relative increase in psychotic experiences after sleep restriction. Therefore, it could be hypothesized that the effect of sleep disruption on psychotic experiences in a more vulnerable group might be correspondingly more severe.

Besides the theoretical importance of these findings, there are some implications for clinical practice. Firstly, monitoring or treating insomnia in individuals at risk of psychosis may reduce transition to first-episode psychosis. Secondly, sleep problems may be maintaining negative emotional states, which themselves may be maintaining distressing psychotic experiences. Treatment of insomnia in psychosis has recently been shown to be highly effective^12^ and well-accepted by patients.^62^ However, while clinicians recognize the importance of sleep, sleep problems are rarely formally assessed or treated,^63^ and are traditionally considered a consequence of psychosis. The current study shows that sleep problems themselves may be causally relevant to the onset or maintenance of distressing psychotic experiences.

## Supplementary Material

Supplementary material is available at *Schizophrenia Bulletin* online.

## Funding

This study was supported by a Wellcome Trust Strategic Award (098461/Z/12/Z) to the University of Oxford Sleep and Circadian Neuroscience Institute (SCNi). SR is supported by an MRC Doctoral Studentship and a Balliol College Dervorguilla Scholarship (Oxford). DF is supported by an NIHR Research Professorship (RP-2014-05-003).

## Supplementary Material

Supp1_StudyFig_13062017

Supp2_ExpandedMeans_13062017

Supp3_interactions_13062017
