# Supplementary figures and images for: Disrupting Sleep: The Effects of Sleep Loss on Psychotic Experiences Tested in an Experimental Study With Mediation Analysis
Source: Schizophr Bull. 2017 Aug 4;44(3):662–71. doi: 10.1093/schbul/sbx103 (PMC5890488; doi:10.1093/schbul/sbx103)

## Supplementary material 1: Study process
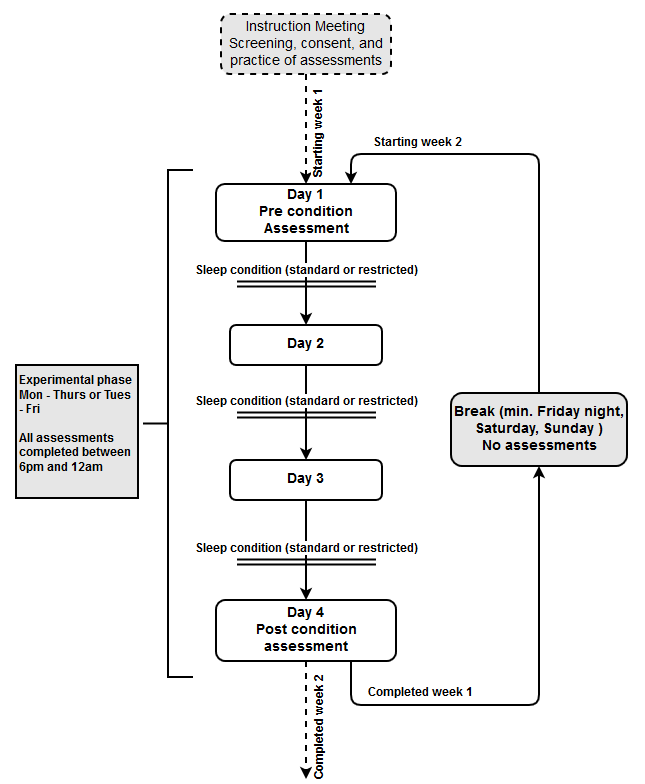

Supplement: Supp1_StudyFig_13062017 [file sbx103_suppl_supp1_studyfig_13062017.doc]
