# Supplementary material for: Disrupting Sleep: The Effects of Sleep Loss on Psychotic Experiences Tested in an Experimental Study With Mediation Analysis
Source: Schizophr Bull. 2017 Aug 4;44(3):662–71. doi: 10.1093/schbul/sbx103 (PMC5890488; doi:10.1093/schbul/sbx103)
Supplement: Supp2_ExpandedMeans_13062017 [file sbx103_suppl_supp2_expandedmeans_13062017.doc]

| **Outcome variable** | **Sleep** | **Order** | **Baseline: mean (SD)** | **Endpoint: mean (SD)** | **Effect (SE), p-value** | **95% CI** | **Effect size** |
| --- | --- | --- | --- | --- | --- | --- | --- |
| Paranoia (SPEQ) | Restricted | Restricted then standard | 2.2 (2.7) | 3.5 (5.2) | 1.57 (.54), 0.003 | 2.63, -0.52 | .383 |
|  |  | Standard then restricted | 2.5 (3.9) | 4.1 (5.6) |  |  |  |
|  |  | Total | 2.3 (3.4) | 3.8 (5.4) |  |  |  |
|  | Standard | Restricted then standard | 1.5 (2.4) | 2.6 (6.7) |  |  |  |
|  |  | Standard then restricted | 4.0 (6.0) | 2.5 (3.9) |  |  |  |
|  |  | Total | 2.8 (4.7) | 2.5 (5.4) |  |  |  |
| Hallucinations (SPEQ) | Restricted | Restricted then standard | .5 (.9) | 1.0 (1.8) | 1.06 (.28). 0.000 | 1.62, -.51 | .869 |
|  |  | Standard then restricted | 2 (.6) | 1.4 (2.9) |  |  |  |
|  |  | Total | .4 (.8) | 1.2 (2.4) |  |  |  |
|  | Standard | Restricted then standard | .2 (.6) | .2 (.4) |  |  |  |
|  |  | Standard then restricted | 1.4 (2.9) | .1 (.8) |  |  |  |
|  |  | Total | .3 (1.5) | .1 (.7) |  |  |  |
| Cognitive Disorganisation (SPEQ) | Restricted | Restricted then standard | 2.4 (3.5) | 4.5 (3.6) | 2.44 (.36), <0.001 | 3.14, -1.73 | .643 |
|  |  | Standard then restricted | 2.7 (4.2) | 5.2 (4.7) |  |  |  |
|  |  | Total | 2.6 (3.9) | 4.8 (4.2) |  |  |  |
|  | Standard | Restricted then standard | 2.3 (3.6) | 2.2 (2.8) |  |  |  |
|  |  | Standard then restricted | 5.2 (4.7) | 2.6 (4.1) |  |  |  |
|  |  | Total | 2.7 (3.8) | 2.5 (3.5) |  |  |  |
| Grandiosity (SPEQ) | Restricted | Restricted then standard | 6.7 (6.1) | 6.7 (6.1) | .14 (.52), 0.780 | 1.16, -0.87 | .002 |
|  |  | Standard then restricted | 6.6 (7.4) | 6.7 (7.2) |  |  |  |
|  |  | Total | 6.6 (6.7) | 6.7 (6.6) |  |  |  |
|  | Standard | Restricted then standard | 7.0 (6.6) | 6.5 (6.2) |  |  |  |
|  |  | Standard then restricted | 6.7 (7.2) | 6.3 (6.8) |  |  |  |
|  |  | Total | 6.4 (6.1) | 6.4 (6.5) |  |  |  |
| Psychotic experience distress (SPEQ) | Restricted | Restricted then standard | .3 (.6) | .7 (1.0) | .42 (.12), 0.001 | 66, -.18 | .521 |
|  |  | Standard then restricted | .4 (.9) | .7 (1.3) |  |  |  |
|  |  | Total | .3 (.8) | .7 (1.1) |  |  |  |
|  | Standard | Restricted then standard | .3 (.8) | .2 (.7) |  |  |  |
|  |  | Standard then restricted | .5 (.9) | .3 (.7) |  |  |  |
|  |  | Total | .4 (.8) | .3 (.7) |  |  |  |

| **Outcome variable** | **Sleep** | **Order** | **Baseline: mean (SD)** | **Endpoint: mean (SD)** | **Effect (SE), p-value** | **95% CI** | **Effect size** |
| --- | --- | --- | --- | --- | --- | --- | --- |
| Depression (DASS) | Restricted | Restricted then Standard | 2.0 (3.4) | 4.5 (5.4) | 2.80 (.61), <0.001 | 3.99, -1.61 | .776 |
|  |  | Standard then restricted | 2.1 (4.5) | 3.7 (6.7) |  |  |  |
|  |  | **Total** | **2.0 (3.9)** | **4.6 (6.1)** |  |  |  |
|  | Standard | Restricted then Standard | 1.5 (3.1) | 1.7 (4.8) |  |  |  |
|  |  | Standard then restricted | 2.1 (3.4) | 1.5 (2.9) |  |  |  |
|  |  | **Total** | **1.8 (3.2)** | **1.6 (3.9)** |  |  |  |
| Anxiety (DASS) | Restricted | Restricted then Standard | 2.6 (3.1) | 5.6 (5.7) | 2.80 (.61), <0.001 | 3.99, -1.61 | 1.002 |
|  |  | Standard then restricted | 1.9 (3.3) | 4.9 (4.7) |  |  |  |
|  |  | **Total** | **2.2 (3.2)** | **5.2 (5.2)** |  |  |  |
|  | Standard | Restricted then Standard | 2.4 (3.1) | 2.5 (5.0) |  |  |  |
|  |  | Standard then restricted | 3,4 (4.3) | 1.7 (2.4) |  |  |  |
|  |  | **Total** | **3.0 (3.8)** | **2.1 (3.9)** |  |  |  |
| Stress (DASS) | Restricted | Restricted then Standard | 3.2 (3.6) | 8.4 (8.6) | 4.61,(.85), <0.001 | 6.27, -2.95 | .806 |
|  |  | Standard then restricted | 4.2 (8.0) | 7.1 (7.2) |  |  |  |
|  |  | **Total** | **3.8 (6.2)** | **7.8 (7.8)** |  |  |  |
|  | Standard | Restricted then Standard | 3.1 (4.4) | 2.8 (5.0) |  |  |  |
|  |  | Standard then restricted | 4.9 (5.9) | 3.8 (4.2) |  |  |  |
|  |  | **Total** | **4.0 (5.3)** | **3.3 (4.6)** |  |  |  |
| Negative self beliefs (BCSS) | Restricted | Restricted then Standard | 1.7 (2.0) | 2.4 (3.1) | 1.05 (.28), <0.001 | 1.60, -.50 | .463 |
|  |  | Standard then restricted | 1.8 (2.6) | 2.8 (3.3) |  |  |  |
|  |  | **Total** | **1.8 (2.2)** | **2.6 (3.2)** |  |  |  |
|  | Standard | Restricted then Standard | 1.5 (2.2) | 1.4 (2.0) |  |  |  |
|  |  | Standard then restricted | 2.2 (2.3) | 1.8 (2.3) |  |  |  |
|  |  | **Total** | **1.9 (2.3)** | **1.6 (2.1)** |  |  |  |
| Positive self beliefs (BCSS) | Restricted | Restricted then Standard | 15.1 (4.4) | 13.7 (4.2) | -0.55 (.53), 0.305 | .50, -1.59 | .113 |
|  |  | Standard then restricted | 11.3 (5.6) | 10.5 (5.4) |  |  |  |
|  |  | **Total** | **13.1 (5.3)** | **12.0 (5.1)** |  |  |  |
|  | Standard | Restricted then Standard | 14.6 (4.6) | 14.0 (5.4) |  |  |  |
|  |  | Standard then restricted | 12.8 (4.0) | 11.9 (5.0) |  |  |  |
|  |  | **Total** | **13.7 (4.4)** | **13.0 (5.3)** |  |  |  |

| **Outcome variable** | **Sleep** | **Order** | **Baseline: mean (SD)** | **Endpoint: mean (SD)** | **Effect (SE), p-value** | **95% CI** | **Effect size** |
| --- | --- | --- | --- | --- | --- | --- | --- |
| Negative beliefs about others (BCSS) | Restricted | Restricted then Standard | 1.4 (2.0) | 2 (3.1) | 0.99 (.36), 0.006 | 1.68, -.29 | .363 |
|  |  | Standard then restricted | 2.5 (3.1) | 3.3 (4.1) |  |  |  |
|  |  | **Total** | **1.9 (2.7)** | **2.6 (3.7)** |  |  |  |
|  | Standard | Restricted then Standard | 1.7 (2.1) | 1.7 (2.2) |  |  |  |
|  |  | Standard then restricted | 2.3 (3.3) | 1.7 (2.6) |  |  |  |
|  |  | **Total** | **2.0 (2.8)** | **1.7 (2.4)** |  |  |  |
| Positive beliefs about others (BCSS) | Restricted | Restricted then Standard | 13.6 (4.2) | 13.5 (3.3) | 0.19 (.56), 0.737 | 1.28, -0.91 | .044 |
|  |  | Standard then restricted | 10.5 (5.1) | 10.1 (5.6) |  |  |  |
|  |  | **Total** | **12 (5.0)** | **11.8 (4.9)** |  |  |  |
|  | Standard | Restricted then Standard | 13.2 (2.5) | 13.2 (4.3) |  |  |  |
|  |  | Standard then restricted | 12.5 (3.8) | 10.9 (5.4) |  |  |  |
|  |  | **Total** | **12.9 (3.7)** | **12.1 (5.0)** |  |  |  |
| Worry | Restricted | Restricted then Standard | 5.7 (6.0) | 6.7 (6.9) | 2.59 (.92), 0.005 | 4.39, -.80 | .402 |
|  |  | Standard then restricted | 6.1 (6.8) | 8.4 (7.7) |  |  |  |
|  |  | **Total** | **5.9 (6.4)** | **7.6 (7.3)** |  |  |  |
|  | Standard | Restricted then Standard | 4.4 (5.1) | 3.5 (5.1) |  |  |  |
|  |  | Standard then restricted | 7.6 (7.5) | 6.5 (6.4) |  |  |  |
|  |  | **Total** | **6.0 (6.6)** | **5.0 (6.0)** |  |  |  |
| Ebbinghaus- difference | Restricted | Restricted then Standard | 79.8 (19.6) | 81.6 (18.3) | 0.74 (1.19), 0.538 | 3.06, -1.60 | .088 |
|  |  | Standard then restricted | 81.4 (17.9) | 76.8 (26.1) |  |  |  |
|  |  | **Total** | **80.6 (18.6)** | **79.1 (22.6)** |  |  |  |
|  | Standard | Restricted then Standard | 80.9 (19.8) | 75.4 (21.2) |  |  |  |
|  |  | Standard then restricted | 81.9 (17.5) | 81.0 (21.4) |  |  |  |
|  |  | **Total** | **81.4 (18.5)** | **78.2 (21.3)** |  |  |  |
| Perceptual vigilance – average RT | Restricted | Restricted then Standard | 425.1 (93.0) | 478.9 (153.1) | 63.13 (56.11), 0.261 | 173.10, -46.84 | .233 |
|  |  | Standard then restricted | 771.7 (1344.0) | 739.7 (524.4) |  |  |  |
|  |  | **Total** | **603.5 (975.3)** | **613.2 (409.9)** |  |  |  |
|  | Standard | Restricted then Standard | 520.1 (354.6) | 515.5 (252.4) |  |  |  |
|  |  | Standard then restricted | 501.7 (242.7) | 561.2 (312.5) |  |  |  |
|  |  | **Total** | **510.7 (300.8)** | **539.0 (283.7)** |  |  |  |

| **Outcome variable** | **Sleep** | **Order** | **Baseline: mean (SD)** | **Endpoint: mean (SD)** | **Effect (SE), p-value** | **95% CI** | **Effect size** |
| --- | --- | --- | --- | --- | --- | --- | --- |
| Perceptual vigilance – no. of lapses  (RT <=500ms) | Restricted | Restricted then Standard | 8.4 (9.7) | 13.8 (12.5) | 2.65 (1.48), 0.072 | 5.56, -0.24 | 0.214 |
|  |  | Standard then restricted | 15.4 (14.7) | 22.2 (17.1) |  |  |  |
|  |  | **Total** | **12.0 (12.9)** | **18.1 (15.5)** |  |  |  |
|  | Standard | Restricted then Standard | 10.8 (9.1) | 13.2 (9.3) |  |  |  |
|  |  | Standard then restricted | 12.7 (13.9) | 17.4 (14.6) |  |  |  |
|  |  | **Total** | **11.1 (11.8)** | **15.4 (12.4)** |  |  |  |
| Decision Value (N-back) | Restricted | Restricted then Standard | .2 (.1) | .2 (.1) | -0.03 (.01), 0.005 | -0.01, -0.04 | . 545 |
|  |  | Standard then restricted | .2 (.1) | .2 (.1) |  |  |  |
|  |  | **Total** | **.2 (.1)** | **.2 (.1)** |  |  |  |
|  | Standard | Restricted then Standard | .2 (.0) | .2 (.1) |  |  |  |
|  |  | Standard then restricted | .2 (.1) | .2 (.1) |  |  |  |
|  |  | **Total** | **.2 (.0)** | **.2 (.1)** |  |  |  |
| No of beads – jumping to conclusions | Restricted | Restricted then Standard | 4.4 (3.8) | 4.2 (3.8) | 0.02 (.27), 0.925 | 0.55, -0.50 | .005 |
|  |  | Standard then restricted | 4.7 (4.4) | 4.9 (5.2) |  |  |  |
|  |  | **Total** | **3.5 (4.1)** | **4.6 (4.5)** |  |  |  |
|  | Standard | Restricted then Standard | 4.4 (4.4) | 4.3 (3.9) |  |  |  |
|  |  | Standard then restricted | 4.0 (2.7) | 4.3 (3.2) |  |  |  |
|  |  | **Total** | **4.2 (3.6)** | **4.3 (3.5)** |  |  |  |
