# Supplementary material for: Disrupting Sleep: The Effects of Sleep Loss on Psychotic Experiences Tested in an Experimental Study With Mediation Analysis
Source: Schizophr Bull. 2017 Aug 4;44(3):662–71. doi: 10.1093/schbul/sbx103 (PMC5890488; doi:10.1093/schbul/sbx103)
Supplement: Supp3_interactions_13062017 [file sbx103_suppl_supp3_interactions_13062017.doc]

**Supplementary material 3: Interaction analysis**

Checking whether interactions between baseline (i.e. at the start of each respective experimental condition) and condition are significant.

SPEQ Paranoia

Interaction term between condition and paranoia = 0.535 (0.156), p=0.001

The graph shows the effect of the interaction and 95% confidence interval across levels of paranoia (x-axis). The y-axis shows the treatment effect on paranoia (comparing restricted sleep to normal sleep).

The interpretation of this is that there is a significant increase in paranoia under restricted sleep compared to normal sleep (because of the positive coefficient). This increase in paranoia is greater as the baseline level of paranoia is higher.

SPEQ Hallucinations

Interaction term between condition and hallucinations = 1.228 (0.280), p<0.001

The graph shows the effect of the interaction and 95% confidence interval across levels of hallucinations (x-axis). The y-axis shows the treatment effect on hallucinations (comparing restricted sleep to normal sleep).

The interpretation of this is that there is a significant increase in hallucinations under restricted sleep compared to normal sleep (because of the positive coefficient). This increase in hallucinations is greater as the baseline level of hallucinations is higher.

SPEQ Cognitive Disorganisation

Interaction term between condition and cognitive disorganisation = -0.06 (0.10), p=0.528

The graph shows the effect of the interaction and 95% confidence interval across levels of cognitive disorganisation (x-axis). The y-axis shows the treatment effect on cognitive disorganisation (comparing restricted sleep to normal sleep).

The interpretation of this is that there is a significant increase in cognitive disorganisation under restricted sleep compared to normal sleep. This increase in cognitive disorganisation is less as the baseline level of cognitive disorganisation is higher.

SPEQ Total Distress

Interaction term between condition and distress = 0.388 (0.154), p=0.012

The graph shows the effect of the interaction and 95% confidence interval across levels of distress (x-axis). The y-axis shows the treatment effect on distress (comparing restricted sleep to normal sleep).

The interpretation of this is that there is a significant increase in distress under restricted sleep compared to normal sleep (because of the positive coefficient). This increase in distress is greater as the baseline level of distress is higher.
